# Increasing miR-126 Can Prevent Brain Injury after Intracerebral Hemorrhage in Rats by Regulating ZEB1

**DOI:** 10.1155/2022/2698773

**Published:** 2022-04-30

**Authors:** Yue Liu, Chuangqi Mo, Xiaoman Mao, Ming Lu, Li Xu

**Affiliations:** Departmennt of Neurosurgery, PuKou Branch Hospital of Jiangsu Province Hospital (Nanjing Pukou Central Hospital), Nanjing 211800, Jiangsu, China

## Abstract

**Background:**

Studies have found that microRNA (miR) is abnormally expressed in intracerebral hemorrhage (ICH) and is considered a therapeutic target for ICH.

**Objective:**

To investigate the expression and role of miR-126 in the ICH rat model.

**Methods:**

The ICH rat model was established, and miR-126 agomir and ZEB1 antagomir were injected into the lateral ventricle of ICH rats. The neurological function and water content of brain tissue were evaluated 48 hours later. Brain tissue around the hematoma of rats was taken to detect the expression of miR-126, ZEB1, glial fibrillary acidic protein (GFAP), and inflammatory cytokines (TNF-*α*, IL-1*β*, and IL-6). The luciferase reporter gene was applied to analyze the relationship between miR-126 and ZEB1.

**Results:**

miR-126 was downregulated in the ICH rat model, while ZEB1 was upregulated. miR-126 agomir or ZEB1 antagomir injection could improve neurological function and cerebral edema in ICH rats. In addition, it could also reduce the expression of TNF-*α*, IL-1*β*, IL-6, and GFAP in the brain tissue of ICH rats. Luciferase reporter gene showed that ZEB1 could be targeted and regulated by miR-126.

**Conclusion:**

miR-126 is downregulated in ICH rats, and miR-126 can reduce brain injury in ICH rats by inhibiting ZEB1 expression.

## 1. Introduction

Intracerebral hemorrhage (ICH) is the most severe subtype of stroke, with high morbidity and mortality [1]. ICH is a disease with an extremely high fatality rate. Even if the patients survive after the onset of the disease, they will have various brain injuries after ICH, which poses a serious threat to people's lives and health. Studies have found that microRNA (miR) is abnormally expressed in intracerebral hemorrhage (ICH) and is considered a therapeutic target for ICH. After ICH, the integrity of cerebral vessels will be destroyed, resulting in ischemia and impaired neuronal function [2]. At present, the mechanism of ICH is unclear, and there is no special medical or surgical treatment that can be used to improve the prognosis of patients [3]. Therefore, it is necessary to clarify the mechanism of ICH and find therapeutic targets.

microRNA (miR) is an endogenous short-chain RNA molecule that can regulate various basic biological events, such as cell growth, death, and differentiation [4]. Previous studies have found that a variety of miRs are abnormally expressed in ICH and play an important role. For example, miR-222 expression is upregulated in the ICH mouse model, and its inhibitor can reduce brain water content, nerve injury, and inflammatory factor expression in the model [5]. miR-7 can inhibit the activation of astrocytes by inhibiting the EGFR/STAT3 signaling pathway, thus alleviating brain injury in the ICH rat model [6]. miR-590-5p is downregulated in ICH mice and LPS-induced microglia, and it can reduce brain injury in ICH mice via regulating Peli1 [7]. miR-126 is a member of the miR family, which has been found to play a key role in regulating endothelial cell function, controlling angiogenesis and maintaining vascular integrity [8]. Previous reports have pointed out that miR-126 produces a marked effect on ICH. In ICH patient serum, miR-126 is detected to be downregulated and is closely related to perihematomal edema [9]. One animal study suggests that miR-126 is downregulated in ICH rat models, and its overexpression can reduce brain damage in rats. However, the way in which miR-126 exerts its protective effect is largely unknown. Through the miR target gene prediction website, we found that binding sites exist between transcription factor zincfinger ebox binding homeobox 1 (ZEB1) and miR-126. ZEB1 is a member of the ZEB family, which can participate in basic biological events such as cell migration, invasion and apoptosis [10]. Previous studies have found that miR-126 can affect the biological function changes of osteosarcoma cells by regulating ZEB1 [11]. However, we do not know whether miR-126 can play a role in protecting brain injury after ICH through ZEB1. Therefore, we suspect that miR-126 can also protect against brain injury after ICH by regulating ZEB1.

This study mainly explores the expression and role of miR-126 in the ICH rat model, aiming at making efforts to clarify the pathogenesis of ICH and explore therapeutic targets of ICH.

## 2. Materials and Methods

### 2.1. Source and Feeding of Rats

Male SD rats were purchased from Changsha Tianqin Biotechnology Co., Ltd. and cultured in an animal room with a room temperature of 21–26°C and relative humidity of 51–57%%. They were allowed to feed freely and given natural light. This animal experiment has been approved by the ethics committee of our hospital, and the experimental process complies with the principles of protection and use of experimental animals [12].

### 2.2. ICH Model Establishment [6]

SD rats were randomly divided into sham operation group, model group, miR-126 agomir group, agomir control group, ZEB1 antagomir group, and antagomir control group, with 6 rats in each group.

Rats were anesthetized with an intraperitoneal injection of 1% pentobarbital sodium (20 mg/kg), and then, they were fixed on rat brain stereotaxic (RWD Life Science). The location of needle insertion was determined according to the image displayed by the instrument. The rat scalp was incised, a hole with a diameter of 1 nm (0.2 nm in front of anterior fontanelle and 3 nm on the right) was drilled by burs, and 2 *μ*L (0.20 U/*μ*L) of type VII collagenase was slowly injected into the pale bulb through a microsyringe. In the sham operation group, the same operation was performed, only collagenase was replaced with the same amount of normal saline. After the model was established, another hole was opened in the same way (0.8 mm behind the anterior fontanelle and 1.5 mm to the right). Ten *μ*m of miR-126 agomir, agomir control, ZEB1 antagomir, and antagomir control (dissolved in 5 *μ*L of normal saline) were injected into the lateral ventricle through the small hole. The sham operation group and the model group received the same volume of normal saline.

### 2.3. Neurological Function Evaluation

After 48 hours of modeling, the neurological function of each group was evaluated [13]. The score was 1–18, the normal score was 2–3, and the most serious defect was 18. The higher the score was, the more serious the neurological defect was. After the evaluation was completed, the rats were executed to take brain tissue for subsequent research.

### 2.4. Determination of Water Content in Brain Tissue

The wet and dry mass method was used to detect the water content of the rat brain tissue. The general operation was as follows: the brain tissue was taken out, the meninges, low brain stem, cerebellum, and other parts of the brain tissue were discarded, and the wet mass of the rest tissues was weighed. Then, they were put into a preset 105°C constant temperature oven for baking until the mass difference between the two samples was less than or equal to 0.2 mg. Water content (%) = (wet mass − dry mass)/wet mass × 100%.

### 2.5. Dual-Luciferase Reporter

The ZEB1-3′UTR wild type (Wt) and ZEB1-3′UTR mutant (Mut) were established by Lipofectamine™ 2000 kit and transferred to the downstream of a luciferase reporter gene to sequence and identify the constructed plasmid. The plasmid with correct sequencing was cotransfected with miR-126-mimics or miR-NC into HEK293 T cells (a cell with high transfection efficiency and easy culture). The luciferase activity was detected using a dual-luciferase reporter gene detection kit (Solarbio, Beijing).

### 2.6. qRT-PCR Detection

TRIzol kit (Invitrogen, USA) was applied to extract total RNA from brain tissues, and UV spectrophotometer and agarose gel electrophoresis were applied to detect the purity, concentration, and integrity of extracted total RNA. Subsequently, reverse transcription was performed according to the reverse transcription kit (Invitrogen, USA), and amplified by SYBR_Premix ExTaq II (Takara, China). Amplification system: 10 *μ*L of SYBR Premix Ex Taq II (2X), 2 *μ*L of cDNA, 0.8 *μ*L of upstream primers, 0.8 *μ*L of downstream primers, and sterile purified water was supplemented to 20 *μ*L. Amplification conditions: predenaturation at 95°C for 30 s, denaturation at 95°C for 5 s, and annealing and extension at 60°C for the 30s. A total of 40 cycles were performed. U6 was taken as the internal reference of miR-126, and the primers were designed and synthesized by Shanghai Jikai Genechem Co., Ltd. These data were analyzed using 2^−∆∆ct^ [14].

### 2.7. Western Blot Detection

The brain tissues of rats in each group were soaked and washed in PBS solution after cooling, then ground into powder on ice. The total protein in the tissue powder was extracted by RIPA lysis. The protein concentration was detected by the BCA method, adjusted to 4 *μ*g/*μ*L, separated by 12% SDS-PAGE electrophoresis, and transferred to the PVDF membrane. Then, it was dyed with Ponceau S solution, soaked in PBST for 5 min and washed, and sealed with 5% skim milk powder for 2 h. ZEB1 (1 : 1000), glial fibrillary acidic protein (GFAP) (1 : 1000), and *β*-catenin (1 : 1000) primary antibody (Abcam, USA) were added and sealed overnight at 4°C. The primary antibody was removed through a washing membrane, and horseradish peroxidase-labeled goat anti-rabbit secondary antibody (Abcam, USA) 1 : 5000 was added, incubated at 37°C for 1 h, and then rinsed with PBS 3 times, with 5 min each. Excess liquid was absorbed from the membrane with filter paper. ECL was used to illuminate and develop in a dark room. The protein bands were scanned, and the gray value was analyzed in the Quantity One software to calculate the protein expression to be detected.

### 2.8. ELISA Detection

The concentrations of tumor necrosis factor (TNF-*α*), interleukin-1*β* (IL-1*β*), and IL-6 in brain tissues of rats in each group were detected by ELISA. The detection process was conducted in accordance with the instructions of the three kits of IL-1*β*, ELISA, TNF-*α* ELISA, and IL-6 ELISA purchased by Guang Rui Biological Technology Co., Ltd., Shanghai.

### 2.9. Statistical Analysis

In this study, statistical analysis of the collected data was performed using the SPSS18.0 software package, GraphPad 7 package was applied for image rendering. K-S test was used to analyze the distribution of measurement data, in which the normal distribution data were expressed as mean ± standard deviation (mean ± SD). The independent *t*-test was used for comparison between groups. One-way ANOVA was used for intergroup comparison. LSD t-test was used for pair-wise comparison afterwards. Repeated measurement ANOVA was utilized for multiple time points. Bonferroni was used for backtesting. When *P* < 0.05, a statistical difference was indicated.

## 3. Results

### 3.1. miR-126 Is Downregulated in ICH Rat Model, While ZEB1 Is Upregulated

**Figure 1:**
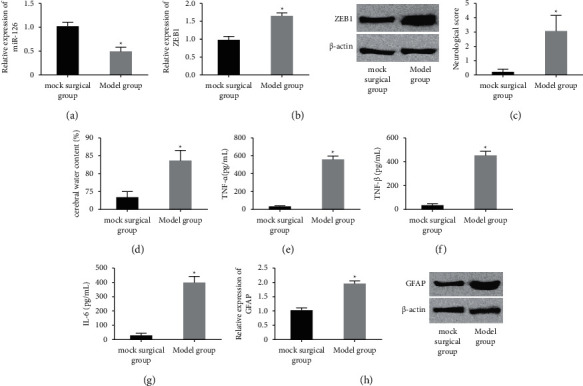
Expression of miR-126 and ZEB1 in ICH rat model. (a) miR-126 was downregulated in ICH rat brain tissue. (b) ZEB1 was upregulated in ICH rat brain tissue. (c) Compared with the sham operation group, the neurological function score in the model group was lower. (d) Compared with the sham operation group, the water content of brain tissue in the model group increased. (e–g) Compared with the sham operation group, the levels of TNF-*α*, IL-1*β*, and IL-6 in the brain tissue of the model group increased. (h) Compared with the sham operation group, GFAP expression in the brain tissue of the model group was increased. Compared with the sham operation group, ^∗^ represents *P* < 0.05.

The expression of miR-126 and ZEB1 in the brain tissues of rats was detected by qRT-PCR and WB, and it was found that miR-126 was downregulated in the tissues, while ZEB1 was increased. The brain injury of rats was evaluated from neurological function, brain tissue water content, brain tissue inflammatory cytokine level, and astrocyte activation. Compared with the sham operation group, the model group had a lower neurological function score, increased brain tissue water content, increased levels of inflammatory cytokines (TNF-*α*, IL-1*β*, and IL-6) and increased expression of astrocyte activation marker GFAP. As shown in Figure 1.

### 3.2. Elevating miR-126 Can Protect Brain Injury in ICH Rats

**Figure 2:**
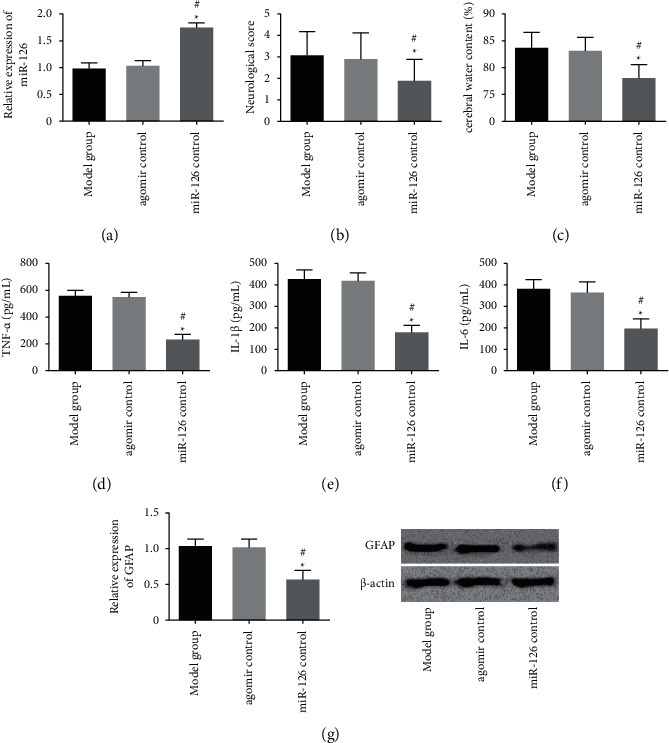
Elevating miR-126 can protect ICH rats from brain injury. (a) Compared with model group and agomir control group, miR-126 in brain tissue of the miR-126 agomir group was decreased. (b) Compared with model group and agomir control group, the neurological function score of the miR-126 agomir group was increased. (c) Compared with model group and agomir control group, water content in brain tissue of the miR-126 agomir group was reduced. (d–f) Compared with model group and agomir control group, TNF-*α*, IL-1*β*, and IL-6 expression in brain tissue of the miR-126 agomir group was reduced. (g) Compared with model group and agomir control group, the expression of GFAP in brain tissue of the miR-126 agomir group was increased. Compared with the model group, ^∗^ represents *P* < 0.05. Compared with the agomir control group, # represents *P* < 0.05.

In order to evaluate the effect of miR-126 on brain injury in ICH rats, we injected miR-126 agomir into the lateral ventricle of ICH rats and observed the brain injury in ICH rats after injection. Compared with the model group and agomir control group, the miR-126 agomir group had a higher neurological function score and lower water content in brain tissue. In addition, the expression levels of TNF-*α*, IL-1*β*, IL-6, and GFAP in brain tissue were lower. This suggested that increasing miR-126 could protect ICH rats from brain injury, as shown in Figure 2.

### 3.3. ZEB1 Is the Downstream Target Gene of miR-126

**Figure 3:**
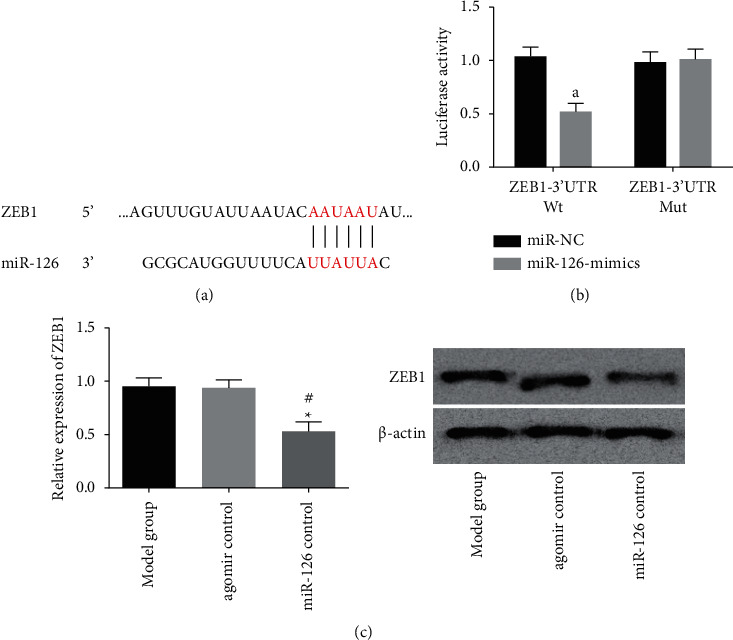
ZEB1 is the downstream target gene of miR-126 (a) There were binding sites between miR-126 and ZEB1. (b) Transfection of miR-126-mimics could inhibit ZEB1-3′UT Wt luciferase activity but would not affect ZEB1-3′UTR Mut luciferase activity. (c) Compared with model group and agomir control group, the expression of ZEB1 in brain tissue of the miR-126 agomir group was reduced. Compared with the model group, ^∗^ represents *P* < 0.05. Compared with the agomir control group, # represents *P* < 0.05. Compared with miR-NC, a represents *P* < 0.05.

The binding sites between miR-126 and ZEB1 were found through biological website prediction. In order to prove the relationship between the two, we carried out dual-luciferase activity detection. The detection revealed that miR-126-mimics transfection can inhibit ZEB1-3′UT Wt luciferase activity but would not affect ZEB1-3′UTR Mut luciferase activity. Subsequently, the expression of ZEB1 in brain tissues of rats in each group was detected by WB, and we found that the injection of miR-126 agomir reduced ZEB1 in tissues. This indicated that ZEB1 was the downstream target gene of miR-126. As shown in Figure 3.

### 3.4. Lowering ZEB1 Can Protect ICH Rats from Brain Injury

**Figure 4:**
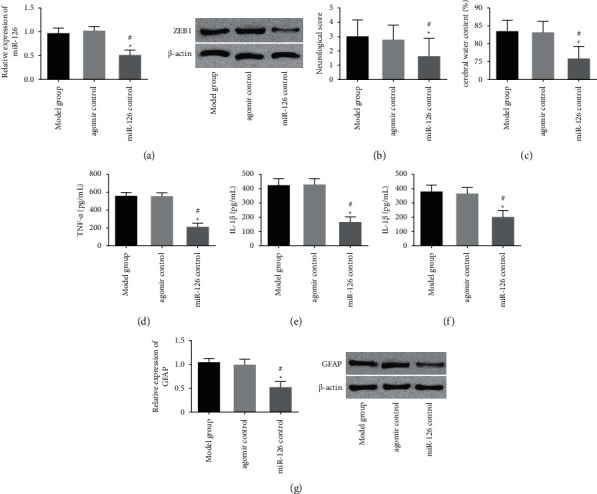
Lowering ZEB1 can protect ICH rats from brain injury. (a) miR-126 in brain tissue of the ZEB1 antagomir group was lower than that of the model group and antagomir control group. (b) Compared with the model group and the antagomir control group, the neurological function score of the ZEB1 antagomir control group was higher. (c) Compared with the model group and the antagomir control group, the water content of brain tissue in the ZEB1 antagomir control group was reduced. (d–f) TNF-*α*, IL-1*β*, and IL-6 expressions in brain tissue of the ZEB1 antagomir group compared with the model group and antagomir control group were reduced. (g) Compared with the model group and antagomir control group, the expression of GFAP in brain tissue of the ZEB1 antagomir group was increased. Compared with the model group, ^∗^ represents *P* < 0.05. Compared with the antagomir control group, # represents *P* < 0.05.

In order to understand whether miR-126 can protect ICH rats from brain injury by regulating the expression of ZEB1, we evaluated the effect of ZEB1 on brain injury in ICH rats. Compared with the model group and the antagomir control group, the ZEB1 antagomir group had a higher neurological function score, lower water content in brain tissue, lower levels of inflammatory cytokines (TNF-*α*, IL-1*β*, and IL-6), and lower GFAP expression. Combined with all previous results, miR-126 could protect ICH rats from brain injury by inhibiting ZEB1 expression, as shown in Figure 4.

## 4. Discussion

Intracerebral hemorrhage (ICH) is the most severe subtype of stroke, with high morbidity and mortality. ICH is a disease with an extremely high fatality rate. Even if the patients survive after the onset of the disease, they will have various brain injuries after ICH, which poses a serious threat to people's lives and health [15]. However, up to now, the pathogenesis of ICH is not known clinically and there is a lack of effective treatment. miR is a ubiquitous RNA in the human body. Lots of miRs are abnormally expressed in human diseases and affect the occurrence and progress of these diseases [16, 17]. In this study, we analyzed the expression of miR-126 in ICH rats and its related effects.

miR-126 is considered an important regulator of physiological angiogenesis and can participate in the pathogenesis of various cardiovascular and cerebrovascular diseases. For example, some studies have found that miR-126 can alleviate acute myocardial ischemia injury through the expression of exosomes derived from adipose-derived stem cells [18]. Other studies suggest that miR-126-5p promotes endothelial cell growth and restricts atherosclerosis by inhibiting Dlk1 [19]. ICH is a vascular disease, miR-126 may play a key role in this disease. After ICH, various brain injuries, such as cerebral edema and neurological deficits, will be caused, which not only poses a threat to the patient's declared health but also increases the redundant medical burden. The results showed that miR-126 was downregulated in ICH rats, and upregulating its expression could improve neurological function and cerebral edema after ICH rats. Astrocytes are the most abundant cells in the central nervous system. After ICH, the cells are rapidly activated, and activated astrocytes secrete a variety of inflammatory factors, thus causing secondary damage to brain tissue [20, 21]. GFAP is a specific molecular marker for the activation of astrocytes [22]. Our research results indicate that up-regulation of miR-126 can reduce the expression of GFAP and inflammatory cytokines in ICH rat brain tissue, suggesting that miR-126 can protect ICH rats from brain injury. However, it is still unclear how miR-126 exerts this protective effect.

It is known to all that miR can participate in biological events by regulating target genes [23]. In order to explore the pathway through which miR-126 plays a protective role in ICH, a miR target gene prediction website was adopted. It was found that there were binding sites between ZEB1 and miR-126, and increasing miR-126 expression could reduce ZEB1 expression. In addition, through the dual-luciferase report, we found that transfection of miR-126-mimics could inhibit ZEB1-3′UTR Wt luciferase activity, but would not affect ZEB1-3′UTR Mut luciferase activity, indicating that miR-126 regulates ZEB1 in a targeted manner. ZEB1 is a zinc finger transcription factor that can participate in biological events such as cell growth, apoptosis and embryo development [24]. At present, reports on ZEB1 in diseases are mainly focused on tumors and it is considered an oncogene [25, 26]. Therefore, alleviating brain injury after ICH is an important part of improving the prognosis of patients. In this study, we analyzed the expression of miR-126 after ICH and its related effects. A variety of miRs have been found to participate in the development of diseases through targeted regulation of ZEB1 expression. For example, in esophageal squamous cell carcinoma, miR-128-3p can inhibit epithelial-mesenchymal transition (EMT) and metastasis of cancer cells by regulating ZEB1 [27]. In cervical cancer, miR-211 can inhibit cancer by inhibiting the expression of ZEB1 [28]. In colorectal cancer, miR-873-5p can target ZEB1 to inhibit the migration, invasion and EMT of cancer cells [29]. The relationship between miR-126 and ZEB1 has been previously reported. Their reports point out that miR-126 can affect the biological functions of osteosarcoma and cervical cancer cells by regulating the expression of ZEB1, thus participating in the development of tumors [11, 30]. However, we do not know whether miR-126 can play a role in protecting brain injury after ICH through ZEB1. Our results show that ZEB1 is increased in ICH rats. In addition, ZEB1 can not only aggravate the neurological function and cerebral edema of ICH rats but also increase the expression of GFAP and inflammatory cytokines in ICH rats' brain tissue. It is suggested that inhibition of ZEB1 may be a therapeutic target for brain injury after ICH. Combined with all the above results, miR-126 can at least partially protect ICH rats from brain injury by inhibiting ZEB1.

## 5. Conclusion

In this study, we have proved that miR-126 can reduce brain injury in ICH rats by inhibiting the expression of ZEB1, but there are some deficiencies. First of all, this article only explores the expression and function of miR-126 and ZEB1 in ICH rats and does not analyze the clinical effect of the two. Secondly, this article only analyzed the role of the miR-126/ZEB1 axis in ICH and has not explored whether miR-126 can play a role in ICH through other target genes. The abovementioned deficiencies are expected to be supplemented in future research. This is our future research direction.

To sum up, miR-126 is downregulated in ICH rats, and miR-126 can reduce brain injury in ICH rats by inhibiting ZEB1 expression.

## Data Availability

The datasets used and/or analyzed during the current study are available from the corresponding author on reasonable request.
